# Nanocomposites Derived from Construction and Demolition Waste for Cement: X-ray Diffraction, Spectroscopic and Mechanical Investigations

**DOI:** 10.3390/nano14100890

**Published:** 2024-05-20

**Authors:** Roxana Rada, Daniela Lucia Manea, Andrzej Nowakowski, Simona Rada

**Affiliations:** 1 Department of Civil Engineering and Management, Faculty of Civil Engineering, Technical University of Cluj-Napoca, 400020 Cluj-Napoca, Romania; roxana.rada@ccm.utcluj.ro (R.R.); daniela.manea@ccm.utcluj.ro (D.L.M.); 2 Strata Mechanics Research Institute of the Polish Academy of Sciences, 30-059 Krakow, Poland; 3 National Institute of Research and Development for Isotopic and Molecular Technologies, 400293 Cluj-Napoca, Romania

**Keywords:** nanocomposites, waste, cement, structure, hardness, strength

## Abstract

In the production of cement, raw materials can be partially substituted by regenerable waste provided from glasses, construction and demolition waste in order to reduce the environmental problem and burden of landfills. In this study, limestone–silicate composites were synthesized using starting materials such as glass waste and lime, brick, autoclaved aerated concrete (ACC), mortar or plaster waste. The structure and mechanical properties of the nano-composite materials have been studied. The mean CaCO_3_ crystallite sizes are higher for composites containing ACC and brick than for doping with lime, mortar and plaster. Cement-based materials are formed by replacing 2.5% of the Portland cement with limestone–silicate composites. The results indicate new possibilities for introducing 2.5%of composites in cement paste because they promote the formation of the C-S-H network, which provides strength and long stability for the cement paste. The influence of varied types of mix composites in the expired cement on the initial cracking strain and stress, tensile strength and compressive strength were investigated. The compressive strength values of composite-expired cement specimens are situated between 11.8 and 15.7 MPa, respectively, which reflect an increase from 22.9 up to 63.54% over the compressive strength of expired cement matrix.

## 1. Introduction

In the world, Portland cement manufacturing occupies the third largest industry in terms of energy consumers and emits 7% of the total CO_2_ emissions in the atmosphere [1]. At these drawbacks, cement production is faced with the fast exhaustion of primary raw materials, such as the decarbonation of lime. The production of 1 ton of cement requires 2.8 tons of raw materials, which generates about 1 ton of greenhouse gas [2]. The development of other classes of cement-based materials as a viable alternative to a circular economy must be researched in the field, according to the European Union. The use of renewable raw materials such as coal gangue, construction and demolition waste, fly ash, metallurgical waste, and glasses is a solution for a circular economy [3,4,5].

The problem of the cement market becomes urgent because of the search for alternative mineral additives due to environmental issues, including the limitations of carbon dioxide emissions and climate modifications and the decrease in ashes and slags. The studies examine the uses of glass waste in applications, including aggregates for cement concrete or as cement replacements [6,7].Glass seems to be an excellent candidate for the utilization of cement or concrete. This option is a promising approach and an environmentally friendly solution for the improvement in the recycling rate of glass waste. The experimental data show that the incorporation of 2% nano-SiO_2_ or 10% waste glass powder or a hybrid of the two in the mortar can decrease the deterioration behavior of concrete. The deterioration process and the durability of concrete are responsible for the alkali–silica reaction, which can initiate cracks and expands [7]. The studies reported that waste glass powder and nano-SiO_2_ could mitigate alkali–silica reaction problems [7,8]. Finer glass grains (≤300 µm) will not produce cracking and can be used as a suppressor and as supplementary cementitious materials to mitigate alkali–silica reactions [9].

Moreover, a substantial quantity of waste is generated during construction and demolition activities. Construction and demolition waste encompasses many materials, namely metal, glass, wood, plastic, concrete and other types of building waste. The increasing volume of construction and demolition waste resulting from the expansion of urban and rural infrastructure is crucial to incorporate all components of waste in the recycling cycle [10]. This approach helps conserve natural resources, reduce the amount of waste and release landfills.

In the cement industry and concrete systems, the use of other Supplementary Materials as a replacement for Portland cement has been intensively studied in recent years. Studies showed that the substitution of gypsum (calcium sulfate) by up to 25%, using limestone, is possible without any modifications of the properties [11,12].

Portland cement is constituted by clinker, which is a polyphasic material having four main mineral phases, only two of which are calcium silicates, such as Ca_3_SiO_5_ (noted with C_3_S) or alite, Ca_2_SiO_4_ (noted with C_2_S) or belite. The other two are calcium aluminates, namely calcium aluminate Ca_3_Al_2_O_6_ (C_3_A) and calcium alumino-ferrites Ca_2_AlFeO_5_ (C_4_AF).

According to the European standard for common cement, the clinker (95% weight) contains 30–70 wt% of tri-calcium silicate (alite, C_3_S), 15–30 wt% di-calcium silicate (belite, C_2_S), 5–15 wt% of C_3_A and 5–10 wt% of C_4_AF. Calcium sulfates are added, mainly gypsum, CaSO_4_∙2H_2_O or other cementitious materials (5% weight).

During the hydration process, these silicates and aluminates (C_3_S, C_2_S, C_3_A and C_4_AF) react with water and form different products, such as calcium–silicate–hydrate (C–S–H) gel structures, ettringite, Portlandite, calcium monosulfoaluminate and calcium monocarbonate. The C3S silicates give the product of the hydration more rapidly than C_2_S. C_2_S, C_3_A and C_4_AF are characterized by slow reactions of hydration. The C–S–H gel (3CaO_2_∙SiO_2_∙3H_2_O) is the main product responsible for the strength of cement. The Portlandite, Ca(OH)_2_ is unstable and reacts with CO_2_ to form calcium carbonate, which makes the cement brittle, and the brittleness reduces the durability of cement/concrete [13]. The formation of CaCO_3_ can be removed by the transforming of Ca(OH)_2_ to other useful compounds before reacting with the atmospheric CO_2_.

The use of ground limestone, CaCO_3_, as an additive to replace Portland cement was investigated because it serves to reduce CO_2_ emissions and to save energy and natural resources. American and European standards permit inclusion between 6% and 35% of the ground limestone. The disadvantages of concrete having higher volumes of Supplementary Materials are the delayed initial setting and slower early strength development. The advantages of using CaCO_3_ in the cement industry consist of the disposal of waste and partial substitution of gypsum and fine aggregates.

The literature data [14,15] concluded a positive accelerating effect of the finely ground CaCO_3_ addition on the rate of hydration of cement paste and the strength development of concrete. Structural (pore structure and morphological features) and mechanical properties of the hydration products are affected by the partial incorporation of CaCO_3_ into the C-S-H phase. Microhardness and Young’s modulus values are remarkably improved, and the effect increases with dosages of up to 15% [14]. The CaCO_3_may impart enhanced strength development by contributing to the bonding of C-S-H particles [14]. The C-S-H growth was also observed around CaCO_3_ particles.

A negative effect on the physical properties of the material was observed when the amount of CaCO_3_ per mass of cement exceeded 10–15% [16]. Hardened concrete may contain up to 5% CaCO_3_ per mass of cement because it has little effect on the macroscopic performance [17,18].

Various products and waste materials such as fly ash, silica fume and rice husk were tested as additives in the concrete. Finely divided silica fume is known to accelerate the hydration of cement and the C_3_S phase.

In brief, synthesizing a mix containing nano-sized CaCO_3_ and/or nano-sized SiO_2_ as an additive to Portland cement can be suggested because these additives can accelerate the hydration of cement and the C-S-H and C_3_S phases.

This paper is focused on the valorization of glass and construction and demolition waste as raw materials in new cement products. By achieving this aim, the waste will be converted from environmental and economic difficulty into profitable products as added value resources in the formulations of new mixtures. A wet chemical synthesis method at a lower temperature will prepare limestone–silicate composites based on combinations of glass powder with construction and demolition waste, namely lime, brick, ACC, mortar or plaster powder. The structure of nanocomposites was investigated by X-ray analysis (XRD), FTIR (Fourier transform infrared) spectroscopy and UV–Vis (ultraviolet–visible) spectroscopy. The effect of the substitution of the limestone–silicate composite in the validated and expired cement material was studied separately by FTIR spectra and Vickers hardness measurements.

The research relevance and novelty of this work are offered by nanocomposites prepared by a viable technology from construction and demolition waste, which are capable of producing high-performance cement materials. The replacement of cement by nanocomposites produces a concomitant reduction in the environmental impact because it releases much lower CO_2_ amounts, and production synthesis does not include the use of processes at high temperatures.

## 2. Materials and Methods

The glasses powder (20 g) and 10 mL of NaOH solution (1N concentration) were stirred by mechanical agitation in a porcelain capsule at 40 °C for 10 min. After that, 10 mL of HCl solution (with 1N concentration) was added and stirred again for 10 min. Stoichiometric amounts of construction and demolition waste powders (20 g) such as lime, brick, ACC, mortar or plaster are introduced into the mixture. The notation of the composites is listed in Table 1. The temperature synthesis was increased gradually to 100 °C for 10 min and then to 350 °C for 20 min when solid composites were produced.

This method was also applied to prepare the mixture of composites containing other waste as raw materials, such as iron, lead, steel iron and ash (Table 2).

Control cement material was prepared using gray Portland cement and water. The water:cement ratios were 0.3:1 and 0.4:1 for the validated and expired cement, respectively. For the preparation of composite–cement, 2.5%of the weight of the cement amount was replaced by composites.

The samples were investigated by analyzing X-ray diffraction (Rigaku X ray diffractometer, Hong Kong, China) and SEM, FT-IR spectra, UV–visible absorption spectra and the measurement of Vickers hardness. For FTIR and UV–Vis analysis, Jasco 6200 Fourier Transform Infrared (Tokyo, Japan) and Perkin-Elmer Lambda 45 UV/VIS Spectrometer (Ontario, CA, Canada) were also used. The specimens used for the Vickers tests (Nova 130 microVickers) have a thickness, *h*, of 25 mm, length, *L*, of 62 mm, and a width, *l*, of 42 mm. The specimens were polished with abrasive sandpaper to obtain a flat surface.

## 3. Results and Discussion

The main components of the glasses used in the production of windows are SiO_2_ (72–74%), Na_2_O (11–14%), CaO (6–11%), and other oxides, such as MgO, K_2_O, and Fe_2_O_3_ in according with literature data [19,20]_._

### 3.1. X-ray Diffraction (XRDData of the Limestone–Silicate Nanocomposite

**Figure 1:**
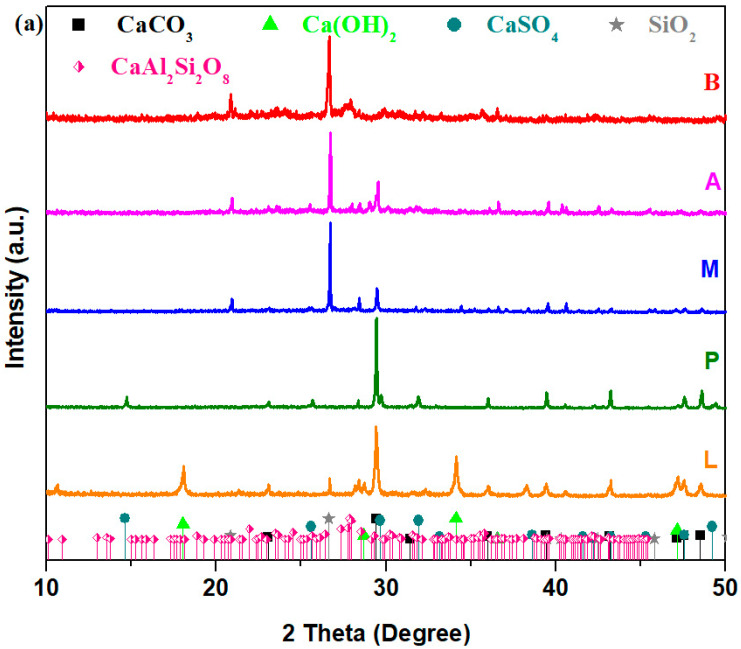

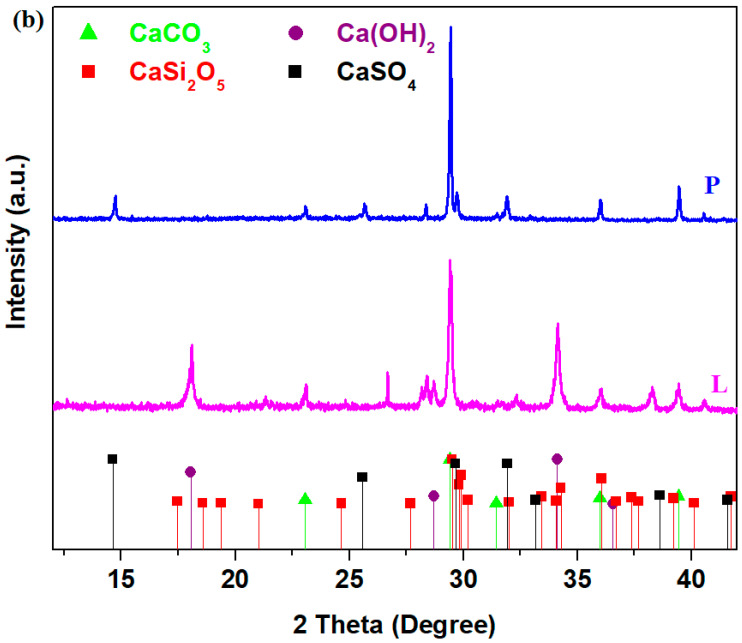
Diffractograms of the CaCO_3_–silicate composites: (**a**) B, A, M, P and P; (**b**) P and L samples.

XRD data of prepared composites are illustrated in Figure 1. XRD patterns of silicate composites doped with mortar and ACC noted with M and A show the presence of SiO_2_ with a hexagonal structure and CaCO_3_ crystalline phase with a rhombohedral structure. Composite B, obtained by adding brick in the silicate glassy, shows the formation of SiO_2_, CaAl_2_Si_2_O_8_·4H_2_O and CaCO_3_ crystalline phases. The composite P consists of CaCO_3_ and CaSO_4_ crystalline phases. In the composite L containing lime, the presence of CaCO_3_ and Ca(OH)_2_ crystalline phases were identified. Composites L and P were also noticeable through the presence of small amounts of the CaSi_2_O_5_ crystalline phase.

The addition of composites in cement material can produce the formation of a C–S–H network because silica—SiO_2_—present in composites reacts with calcium hydroxide present in cement in accordance with the following reaction:

3 Ca(OH)_2_ + 2 SiO_2_ → 3CaO∙2SiO_2_∙3H_2_O   (or C–S–H gel)

The median particle size (D) can be determined by the Debye–Scherer equation [17]:

$$D=\frac{0.94\;\lambda }{\beta cos\theta }$$

where λ is the X-ray wavelength (0.154 nm), β is the broadening of the diffraction peak in radians (full width at half maximum of the peak), and θ is the diffraction angle for the maximum peak in radians.

The median particle size of the main diffraction peaks for prepared composites with varied dopant types obtained using the Debye–Scherer equation is shown in Table 1. The median particle size has the lowest value for the silicate composites containing lime and the highest value for the brick–silicate composites.

The data shows that the CaCO_3_ median crystallite sizes were 63.71 and 95.55 nm in composites L and M, and they increased from 119.7 to 261 nm in composites A and B, respectively.

The values of CaCO_3_ crystallite sizes from the limestone–silicate composite increased in ascending order as follows: L < M < P < A < B. Increasing order of SiO_2_ particle sizes, namely M < A < B, was also observed. The particle sizes in the lime composites have lower values. It is known in the cement structure that the smaller particle sizes improve the pore structure while the higher-sized particles can modify the porosity and transport characteristics at later ages [21].

### 3.2. Scanning Electron Microscopy (SEM) of the CaCO_3_–Silicate Nanocomposites

**Figure 2:**
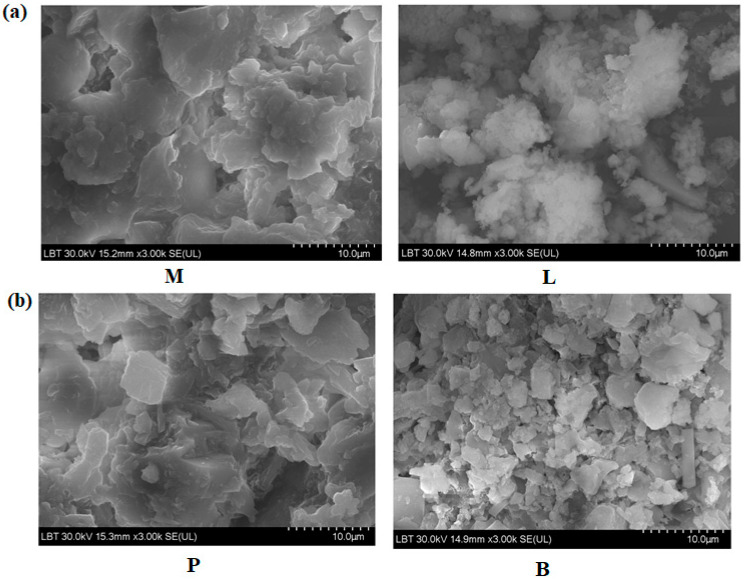
SEM micrographs of prepared composites. The scale bar was 10 µm. (**a**) M and L composites and (**b**) P and B composites.

Further, the characterization of nanocomposites was performed using SEM analysis. SEM micrographs of the CaCO_3_–silicate composites are illustrated in Figure 2.For the sample containing lime, evidence of smaller crystallites and lower structural disorder was observed, confirming the XRD results. Samples doped with mortar and plaster exhibited a higher crystal density than samples containing brick. SEM images of brick–silicate composite reveal random aggregates and larger pieces that have irregular shapes. For the composites containing mortar and plaster, SEM micrographs show larger pieces of the silicate network.

### 3.3. Structural Investigations of CaCO_3_–Silicate Compositesby Fourier Transform Infrared Spectra

**Figure 3:**
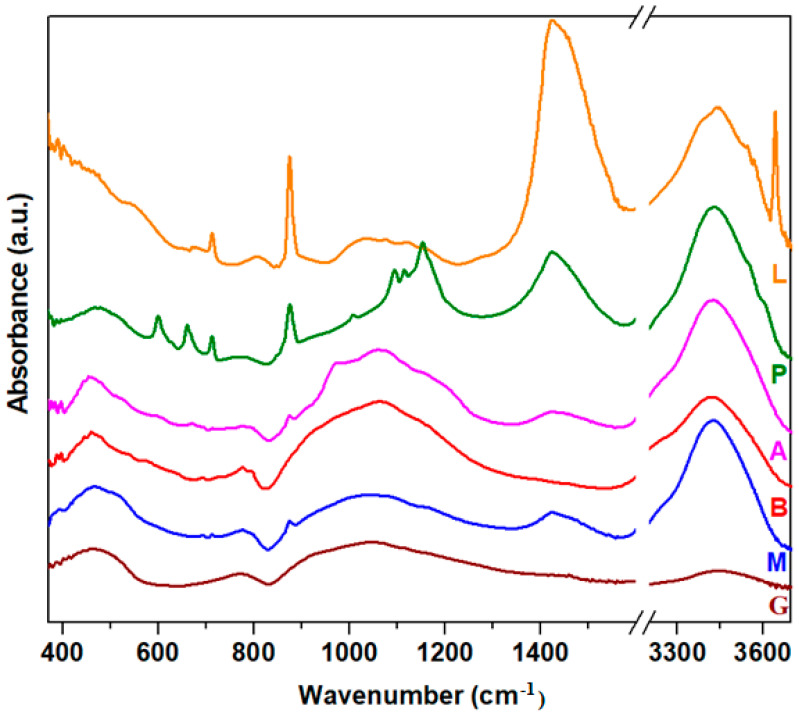
FTIR spectra of studied composites.

FTIR spectra of silicate nanocomposites are shown in Figure 3. The analysis of the IR spectrum of the waste glass indicates some specific regions.

The first region of IR bands situated between 370 and 550 cm^−1^ corresponds to the bending vibrations of the Si-O-Si angles. For the L composite, the intensities of IR bands are more intense than the intensity observed in glassy or other composites.

The second region of the bands with prominent intensity located between 900 and 1300 cm^−1^ is attributed to the stretching vibrations of Si-O bonds in the [SiO_4_] tetrahedral units. For the samples L and P doping with lime and plaster of the glassy network, the intensity of the last bands decreased, while for adding mortar, ACC and brick, an increasing trend of strength was evident. According to the XRD data, the main content of the M, A and B composites is the SiO_2_ (quartz) crystalline phase. The quartz present in composites is utilized during the setting of the cement to produce C-S-H gel.

In silicate systems, the existence of varied distributions of the Q_n_ structures, where *n* is the number of bridging oxygen atoms (n = 0, 1, 2, 3, 4) in the [SiO_4_] structural units, can be characterized in the Si-O matrix. The IR bands corresponding to the Q_0_, Q_1_, Q_2_, Q_3_and Q_4_ units are situated at ~800, 930, 980, 1085 and 1135 cm^−1^ in the FTIR spectra [22,23,24].

For composite A containing ACC, the increase in intensity of the IR bands centered at approximately 980, 1085 and 1135 cm^−1^ shows the presence of metasilicate (Q_2_), disilicate (Q_3_) and tectosilicate (Q_4_) units, respectively. For composite B containing brick, the intensity of the IR bands situated between 930 and 1135 cm^−1^ indicates the formation of pyrosilicate (Q_1_) and tectosilicate (Q_4_) units. The addition of mortar in the glass network (composite M) shows the apparition of the three IR bands centered at about 930, 1085 and 1135 cm^−1^, corresponding to the pyrosilicate, disilicate and tectosilicate units. The CaAl_2_Si_2_O_8_ and SiO_2_ (quartz) crystalline phases are known to have tectosilicate units, where all oxygen atoms exist as bridging oxygen atoms [25]. In the CaAl_2_Si_2_O_8_ crystalline phase, the tetrahedral [SiO_4_] and [AlO_4_] units exist, and the large charge deficit is compensated by the addition of calcium ions. The SiO_2_ (quartz) crystalline phase has a three-dimensional network formed of interconnected [SiO_4_] tetrahedral units.

By doping with lime, plaster, mortar and ACC (composites noted with L, P, M and A), the presence of two IR bands centered at approximately 875 and 1450 cm^−1^ shows the formation of the CaCO_3_ crystalline phase. The IR band centered at approximately 1450 cm^−1^ is assigned to the asymmetric stretching vibrations of CO_3_^2−^ carbonate ions. A new IR band appears centered at approximately ~875 cm^−1^, corresponding to the out-of-plane bending vibrations of carbonate ions [26]. The intensity of these bands decreases after doping with plaster, mortar and ACC, attains the maximum value for the sample doped with lime, and disappears for the sample with brick.

After doping with plaster, the silicate network presents new characteristic IR bands centered at approximately 980 cm^−1^ (Q_2_ units), 1085 cm^−1^ (Q_3_ units) and 1135 cm^−1^ (Q_4_ units). The intensity of the IR band centered at approximately 980 cm^−1^ is lower compared with its analogs. The doublet of IR bands observed in the region at approximately 605 and 670 cm^−1^ is assigned to the out-of-plane and in-plane bending vibrations of sulfate units. Additionally, the characteristic IR bands at ~1200 cm^−1^ correspond to the formation of the calcium sulfate crystalline phase. For sample L, a new IR band centered at ~800 cm^−1^ indicates the presence of orthosilicate units (Q_0_ units).

Our results show that IR spectra of the silicate host matrix differ from each other by doping with varied construction and demolition waste powder, and a process of depolymerization of the silicate network in Q_n_ silicate units was observed. Through the conversion of the silicate matrix in a new Q_n_ silicate network, the formation of new crystalline phases of the metallic ions, namely calcium ions, was observed. The highest degree of crystallinities was remarked for the composites doped with lime and plaster.

### 3.4. UltraViolet–Visible Spectraand Gap Energy Values of CaCO_3_–Silicate Composites

**Figure 4:**
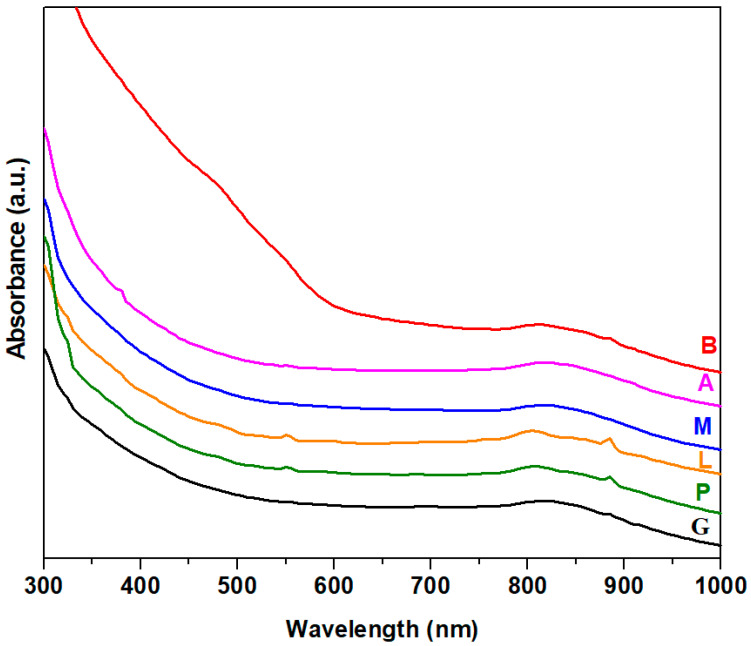
UV–Vis spectra of composite samples.

**Figure 5:**
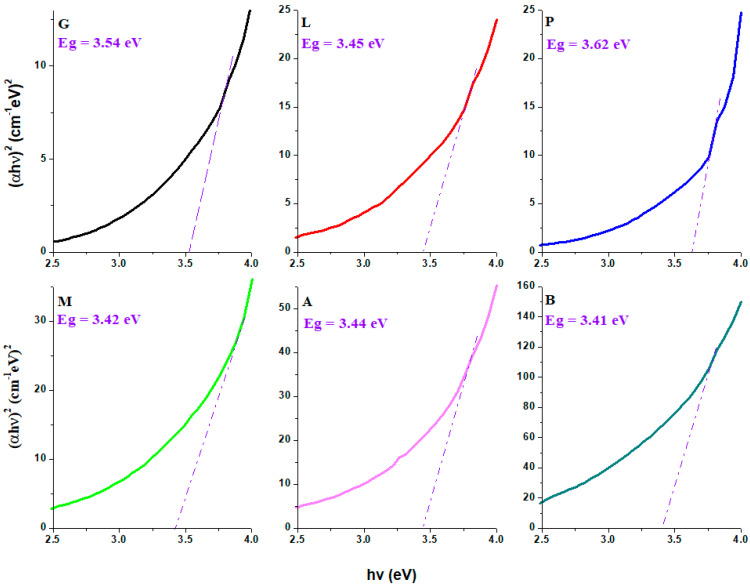
The (*αhν*)^2^ versus *hν* of the limestone–silicate composites. The blue line represents the extrapolation of the gap energy value.

UV–Vis spectra and the plots (*αhν*)^2^ versus *hν* of the nanocomposites are indicated in Figure 4 and Figure 5. The UV–Vis spectra indicate a shift of the absorption edge towards a higher wavelength by doping. For the sample containing brick, the intensity of the UV-Vis absorption suddenly increased, and the absorption edge shifted towards the lower energies more than its analogs. The shift of absorption edge to low energy side occurs when the concentration of non-bridging oxygen atoms and/or defects is increased. The red shift of the absorption edge shows the reduction of the gap energy value.

The values of gap energies, *Eg*, were calculated to be between 3.41 and 3.62 eV. The Eg values of the composites are slightly different from that of standard glass (3.54 eV). By doping with lime, mortar, ACC and brick of the glass network, the values of the gap energies decreased, while the addition of plaster in the host matrix resulted in an increase in the band gap energy. The variations of the optical band gap correspond to the changes in the microstructure of the host matrix. The decrease in Eg value always means the formation of non-bridging oxygen atoms.

For the L, B, M, and A composites containing lime, brick, mortar and ACC, the smaller values of the gap energies can be associated with the presence of non-bridging oxygen ions in the Q_o_, Q_1_ and Q_2_ silicate units according to IR data. In the plaster–silicate composite noted with P, the number of Q_2_ silicate units is smaller compared with composite A (doped with ACC), which results in an increase in E_g_ value. The polymerization degree of silicate units increases in the sample P.

### 3.5. Fourier Transform Infrared Spectra of Composite–Cement Materials

**Figure 6:**
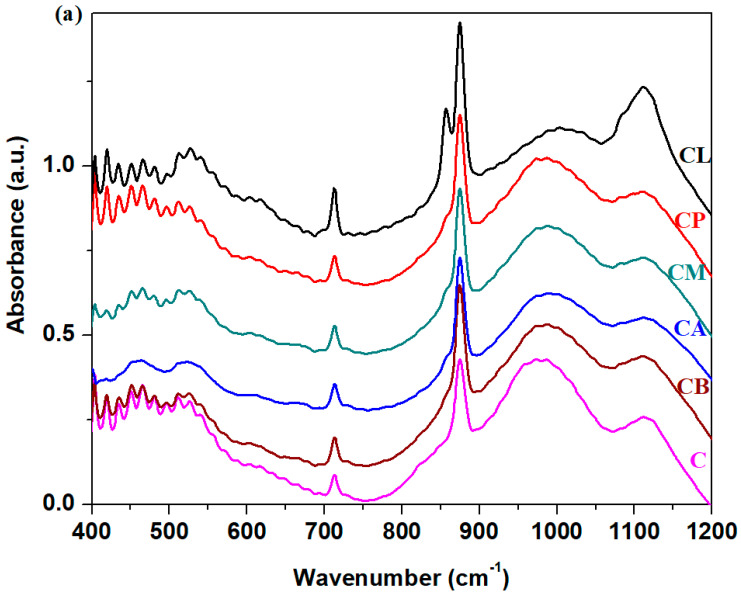

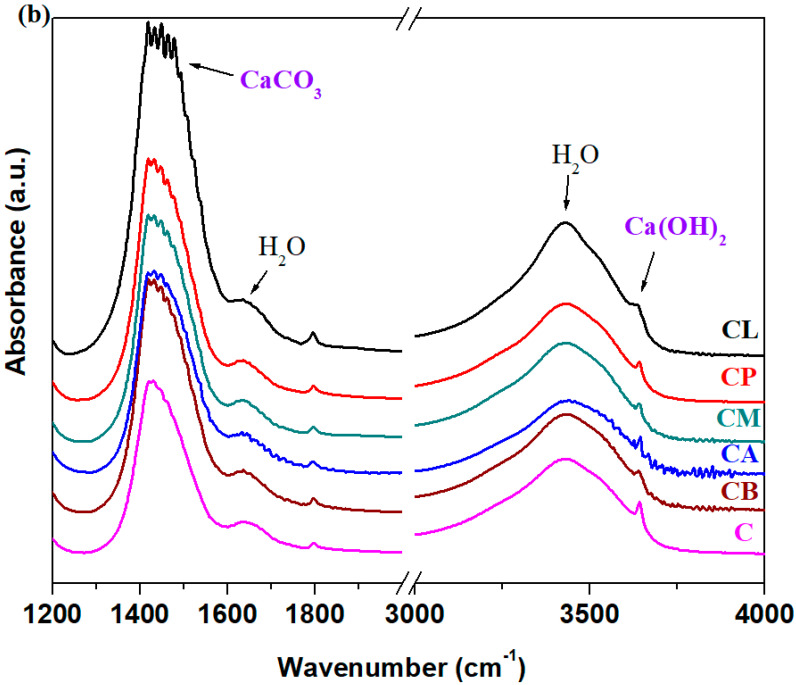
FTIR spectra of composite–cement materials containing Portland cement. (**a**) in the 400–1200 cm^−1^ region and (**b**) in the 1200–4000 cm^−1^ region.

The FTIR spectra of the composites-validated cement materials performed 28 days after their preparation are shown in Figure 6. FTIR spectrum of the reference Portland cement shows some characteristic bands centered at approximately 470, 520, 713, 875, 980, 1115, 1430, 1650, 3420 and 3645 cm^−1^.

The lower intense IR band centered at about 1650 cm^−1^ and broad band centered at about 3420 cm^−1^ can be assigned to the H-O-H bending vibrations and H-O stretching vibrations, confirming the presence of water in the samples. By adding composites to the cement material, the intensities of these vibrations were increased, suggesting the presence of structural water in the reaction product (water bound in the hydrated phases). In practice, the existence of structural water in the composite-validated cement material implies the improvement in the concrete’s hydration processes.

The characteristic vibration bands of the C-O bonds from calcium carbonate are attributed approximately at 713, 875, 1080 and 1425 cm^−1^. For all composite-cement materials, the intensities of these IR bands were enriched, showing the carbonation of samples by the formation of calcium carbonate. The maximum values of intensities of these IR bands were observed in the CL material.

The intense IR band observed at approximately 980 cm^−1^ is typically for the calcium silicate hydrate phase (noted as C–S–H), and the broader band situated in the region between 1080 and 1200 cm^−1^ demonstrates the existence of Si-gel. The position of the first IR band shifts towards larger wave-numbers, and the intensity of the second band was increased by the addition of composites in the cement material. The shape of IR bands located in the range between 900 and 1200 cm^−1^ was modified by doping with composites. These IR bands are assigned to the C–S–H and other alumino-silicate phases from cement material. These evaluations indicate that the crystalline phases formed in the cement have varied microstructures, according to XRD data. For the composite-validated cement materials, a new band appears centered at about 855 cm^−1^, corresponding to the Ca-O bond.

The presence of a sharp band at about 3645 cm^−1^ is an indicator of the formation of Ca(OH)_2_ crystalline phase in cement materials. For the CL material, the intensity of this band increases, attaining a maximum value. The larger amount of Ca(OH)_2_ can be a result of a higher degree of cement hydration and is also correlated with the formation of more C-S-H phases after adding the composite admixtures.

In all limestone composite cement materials, the IR spectra reveal a similar intensity of the band centered at approximately 980 cm^−1^, which confirms that the addition of limestone composites did not delay the formation of C-S-H gel and Ca(OH)_2_. A slight increase in the numbers of carbonate units and water in composite–cement materials was also seen.

**Figure 7:**
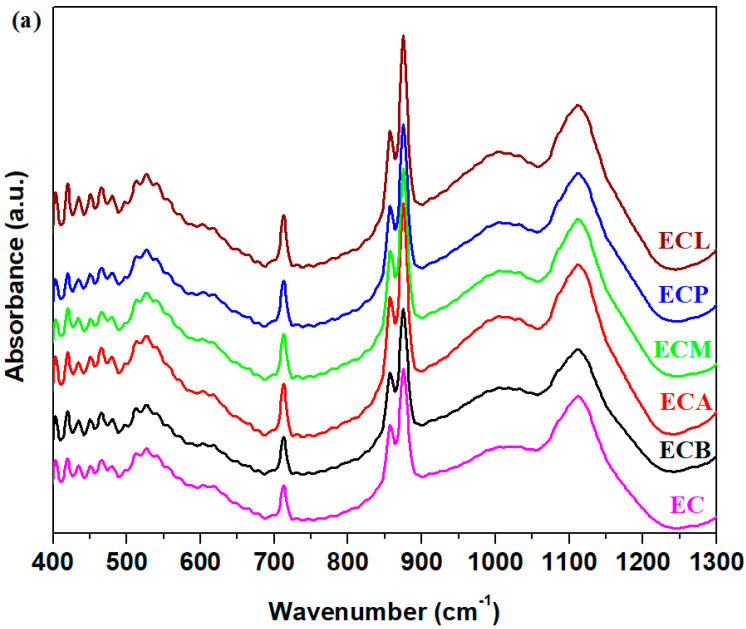

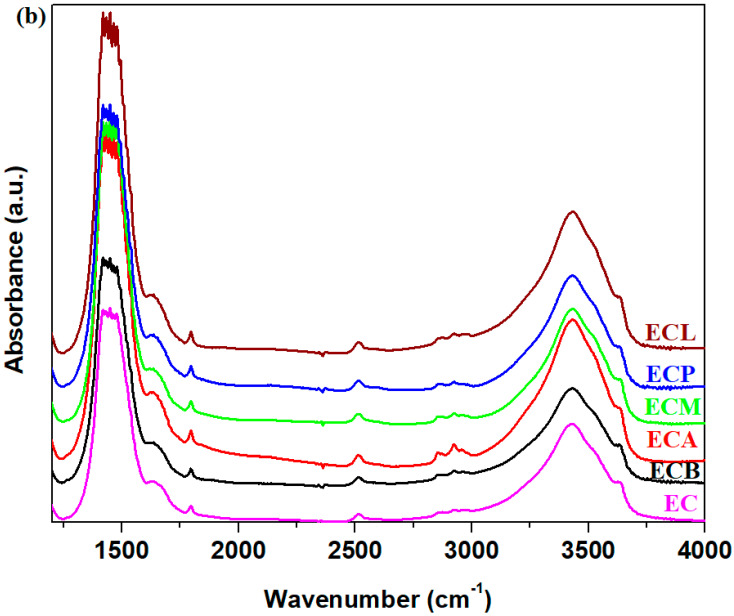
FTIR spectra of composites-expired cement materials. (**a**) in the 400–1300 cm^−1^ region and (**b**) in the 1200–4000 cm^−1^ region.

The FTIR spectra of the CaCO_3_–silicate composites-expired cement materials performed 28 days after their preparation can be observed in Figure 7. An inspection of IR spectra shows that the IR bands situated between 800 and 1200 cm^−1^ were improved gradually in their intensities by the addition of varied composites in the structure of the expired cement materials. The ascending order of increase of intensity of these IR bands is brick < plaster < mortar < lime < ACC when these composites were mixed with expired cement. For the brick composite–cement material, the intensity of these bands was almost the same as that of expired cement. These regions are attributed to the stretching vibrations of the calcium–silicates–hydrates (C–S–H) and silicate units.

The intensities of the IR bands centered at approximately 715 and 1445 cm^−1^, corresponding to C–O vibrations from calcium carbonate, show maximum values for the samples containing mortar, ACC and lime, according to XRD data. The shoulder situated at about 3630 cm^−1^ is attributed to the presence of Ca(OH)_2_ phase in the expired cement increases for all doped samples, except for the addition of brick composite in the cement paste.

The intensity and the position of the IR band centered at about 530 cm^−1^ were affected slightly by the doping with composite in the expired cement mix. This IR band corresponds to the bending vibrations of Si-O out-of-plane.

**Figure 8:**
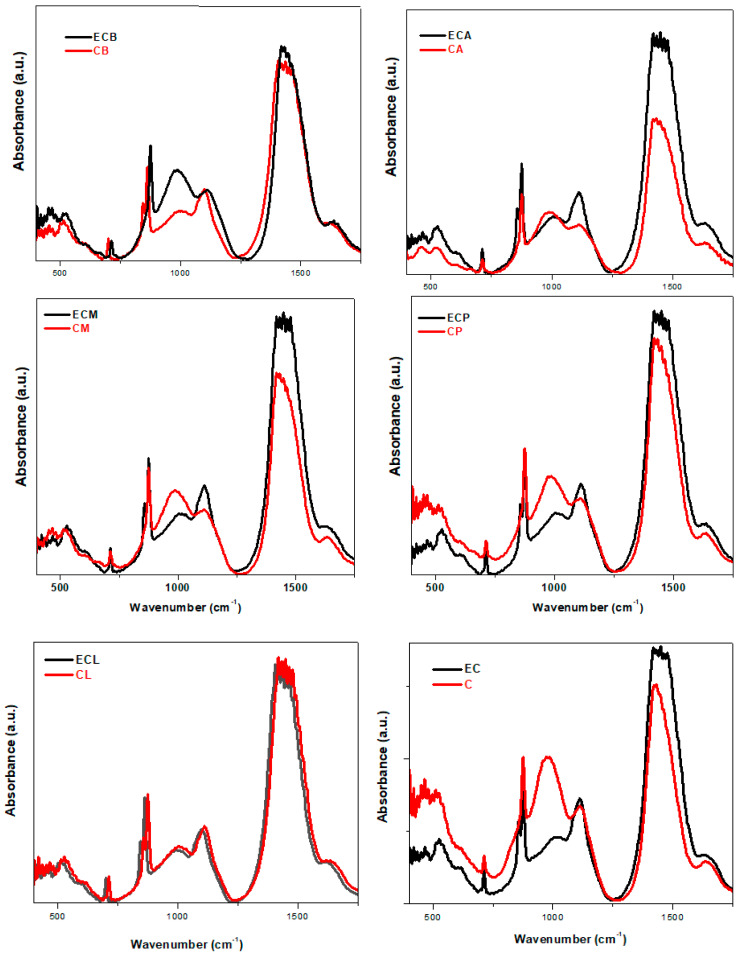
FTIR spectra of composite-validated/expired cement materials.

Figure 8 shows the comparative IR spectra of the composite–cement materials having validated or expired cement their composition. The structural modifications occurring in the expired cement can be easily observed on the IR spectrum in comparison to validated cement. Almost the same tendency in intensity of IR bands was observed in the case of doping with lime in both cement materials. In the other cases, the IR spectrum of composite-validated cement differs significantly from the others with expired cement.

**Figure 9:**
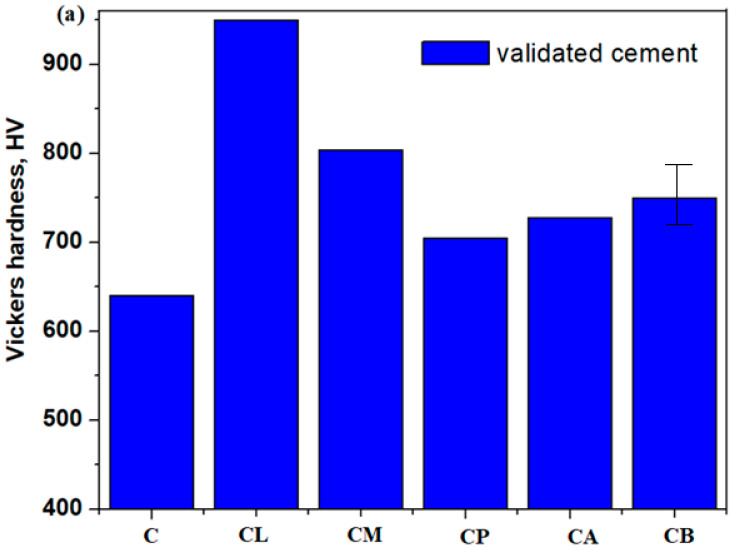

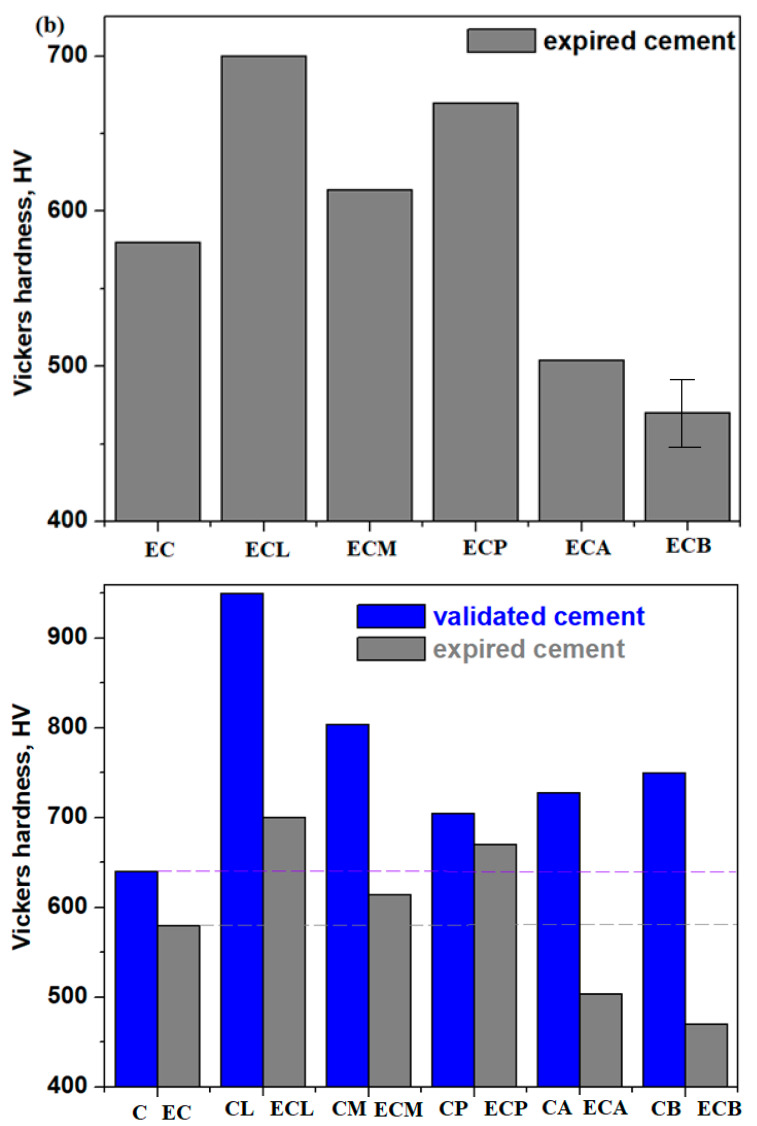
Influence of composite types on the Vickers hardness distribution of (**a**) composite-validated materials and (**b**) expired cement materials. An error of 2% in the Vickers hardness values can be adjusted.

In the brick–composite cement material with expired cement (sample ECB), a downward shift in the intensity of IR bands situated between 400 and 1600 cm^−1^ in comparison to validated cement can be noticed. These structural changes at the micro level have a drastic effect on the mechanical properties (see Figure 9).

By adding the mortar–composite to the cement materials, the IR bands located in the region between 1100 and 1750 cm^−1^ become stronger by using expired cement. These changes correspond to the conversion of C-S-H into modified [SiO_4_] units and the improvement in the amount of the calcium carbonate phase.

The smaller bands in intensity were observed in the region between 400 and 1100 cm^−1^, and the strongest absorption features near 1500 cm^−1^ were observed by the doping of plaster in expired cement. These modifications were referred to as the decrease in the C-S-H phase and the increase in the calcium carbonate phase. For the ECA material, the infrared spectrum shows the existence of intense IR bands assigned to the Si-O-Si bonds and carbonate vibrations bands.

### 3.6. Mechanical Properties of the Limestone Composite–Cement Materials

**Figure 10:**
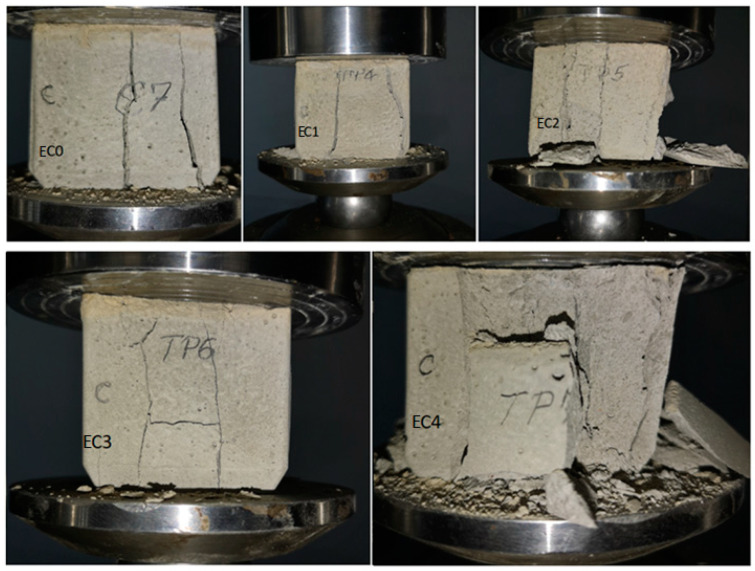
Crack pattern of specimens after failure.

Figure 9 and Figure 10 indicate the effects of varied composites on the Vickers hardness distribution of composite–cement materials using validated and expired Portland cement.

The values of Vickers hardness of the composite–cement materials were higher than those of the validated control cement. Some authors show that the complex IR bands situated between 800 and 1200 cm^−1^ are assigned to the presence of calcium silicate hydrate gel. The formation of C-S-H gel is responsible for the strength of the concrete [27]. The position of the IR band centered at approximately 980 cm^−1^ shifts to 987 cm^−1^ for doping with plaster, mortar, and brick and to 990 and 1005 cm^−1^ for ACC and lime, respectively. The intensities of the IR bands assigned to the Ca-O bonds (~855 cm^−1^) and the presence of Ca(OH)_2_ (~3645 cm^−1^) are also higher than control cement. The calcium ions react with the silicate network and, as a result, form a secondary C-S-H gel. The width and depth of IR bands located in the range between 800 and 1200 cm^−1^ are higher in the case of a composite–cement mix. These compositional evolutions suggest that a higher amount of C-S-H gel results in composite–cement materials and, therefore, a further increase in Vickers hardness. Using limestone composites mixed with validated cement results in a more compact cement material with increased strength.

The values of Vickers hardness of the composite-expired cement materials containing lime, mortar and plaster increase then decline gradually in the presence of brick and ACC.

The IR band centered at approximately 980 cm^−1^ is higher for composite-validated cement mix than for composite-expired cement mix except for the doping with lime (see Figure 8). Therefore, the amount of formed C-S-H gel is small; the strength was decreased by using expired cement.

The smaller value of the Vickers hardness through the doping of expired cement with brick can be understandable because this composite did not contain the CaCO_3_ crystalline phase. This is not surprising because it is well known that the presence of CaCO_3_ in the cement results in an increase in the rate of hydration and the strength of the concrete.

Only the presence of lime or plaster composites (L or P samples) in the cement material, either validated or expired, results in higher values of the Vickers hardness than standard Portland cement. The L and P composites have the presence of CaSi_2_O_5_ crystalline phase in their structure, which can be responsible for the formation of Ca_2_SiO_4_ and Ca_3_SiO_5_ crystalline phases of the cement according to the equation of chemical reaction:

CaSi_2_O_5_ + 4 CaO → Ca_2_SiO_4_ + Ca_3_SiO_5_

The C_3_S and C_2_S silicates are responsible for the strength of the hardened cement paste. The Ca(OH)_2_ amount decreases in these composite–cement materials because it will promote the transformation in the C-S-H matrix, which provides strength and long-term stability to the set cement.

In this study, we observe significant changes in the silicate and carbonate absorption IR bands of composite-expired cement materials. The smaller amount of C-S-H gel (IR band at 970 cm^−1^) and the impoverishment of Si-O-Si bending vibrations (IR band at ~530 cm^−1^) are responsible for the decrease in the Vickers hardness value. The intensity of the highest IR band centered at approximately 1500 cm^−1^ decreases by the doping in the cement materials from lime at the plaster, after that at mortar, ACC and brick. The presence of calcium carbonate crystalline phase accounts for an increase in the Vickers hardness. The size of CaCO_3_ particles is smaller in the cementing materials containing lime or plaster composite, and both with the presence of Ca(OH)_2_ or CaSO_4_ crystalline phase in the cementing materials accounted for an increase in the Vickers hardness. Thus, the presence of SiO_2_ crystalline phase in the cementing materials with mortar-, ACC- or brick-composite led to the decalcification of the cement paste, which then caused micro-cracks of cement hydration products, according to SEM images and a decrease in the Vickers hardness. The IR spectra are used to study the chemistry of prepared cementing materials and their interrelation with mechanical properties.

In brief, the limestone–silicate nano-composites in cementing materials are beneficial as substitutes for cement for construction due to minimizing production cost and pollution. The synthesis temperature used for the production of the cement is approximately 1400 °C with high energy consumption, while in this paper, the synthesis temperature of the composite was 350 °C. Blending cement with recycled composites is more durable and sustainable for environmental impact, and it also reduces energy requirements. The possibility of reintroducing construction and demolition waste back into the life cycle represents significant support for environmental approaches and the release of landfills.

The combination of lime or plaster nano-composite with expired cement produces a structural and mechanical performance that is suitable for standard Portland cement. The use of composites from wastes as a substitute for cement in construction shows some advantages, namely, the conservation of natural resources, the reduction of surface at landfills and CO_2_ emissions in the environment.

Our results indicate that adding 2.5% limestone composites in cement-based materials offer mechanical performance comparable with the cement paste and can also help reduce the CaCO_3_–cement and water–cement ratio in the cement paste.

### 3.7. Mixed Composites Cement Materials

To verify the applicability and feasibility of the synthesis method for composites containing construction and demolition waste in the mixture, four types of composites containing a mixture of construction and demolition waste were prepared. Other construction and demolition waste, such as iron, lead, steel iron and ash powders, were used in the preparation of the composite materials. The detailed preparation of these composites and mechanical properties was described in Ref. [4].

For each composite-expired cement mixture, approximately 50 × 50 × 50 mm prismatic samples were cast and kept in air. Specimens noted with EC1, EC2, EC3 and EC4 containing expired cement and a mix of composites, namely, EC1, have L, G and M composites; EC2 contains L, G, M, A, and B composites; EC3 contains iron, lead, steel iron and ash composites; EC4 composite includes all ten waste (Table 2).

In this study, the mechanical strengths of these specimens were evaluated 7 days after their preparation. The mean values of the specimens of the standard expired cement, notably EC0 and EC1, EC2, EC3 and EC4 mixtures, and the values of compressive strength (*R_c_*) and compressive force are shown in Table 3.

**Figure 11:**
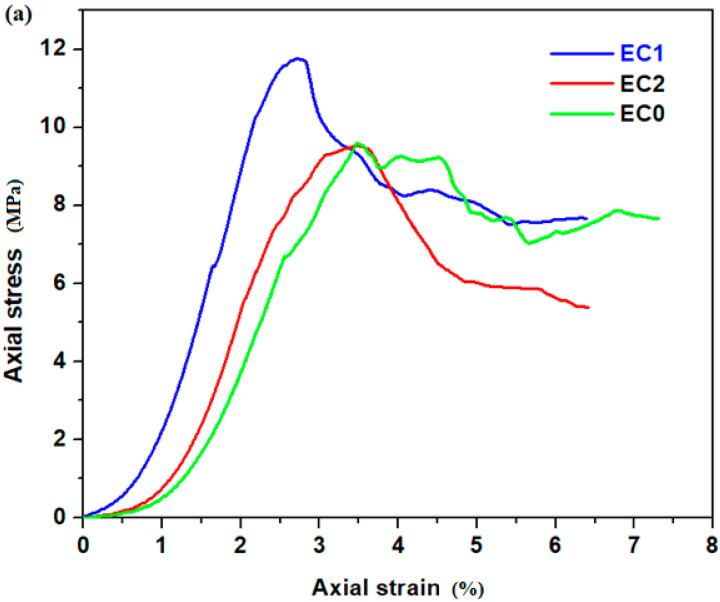

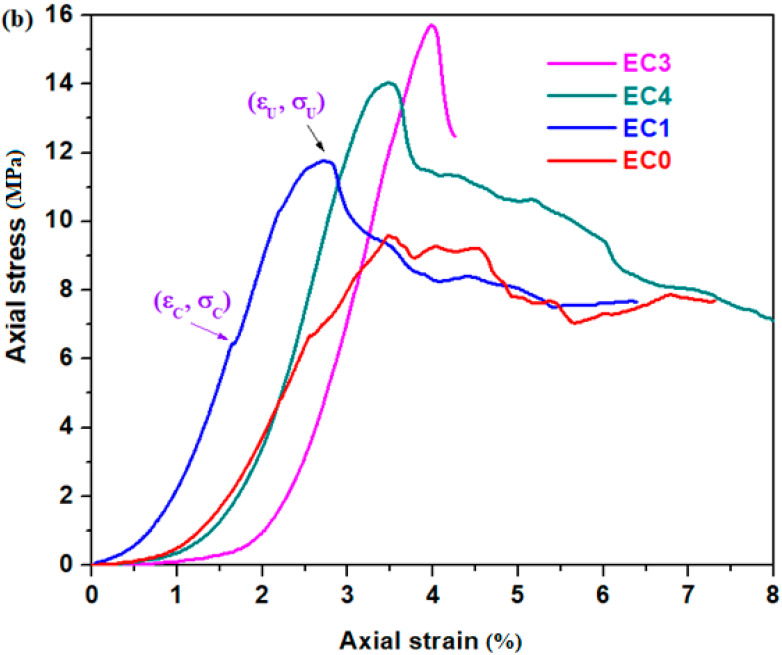
The stress–strain curve of studied specimens. (**a**) EC0, EC1, EC2; (**b**) EC0, EC1, EC3, EC4.

**Figure 12:**
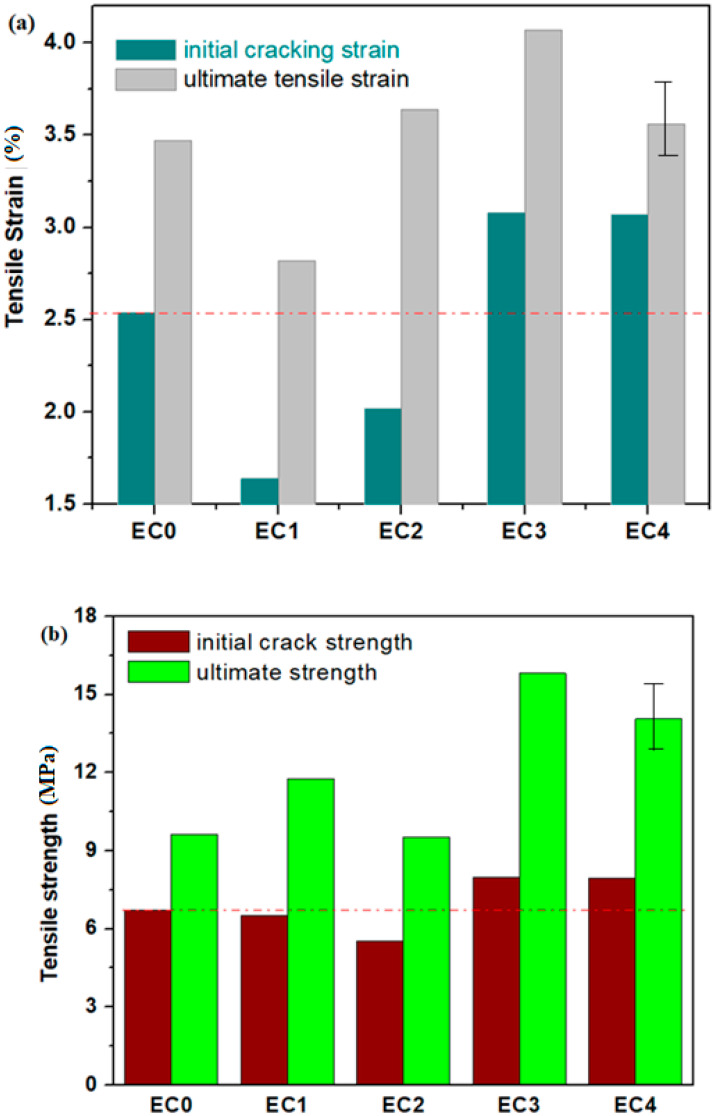
Compositional structure of the composites on uniaxial tensile tests. (**a**) EC0, EC1, EC2, EC3, EC4; (**b**) EC0, EC1, EC2, EC3, EC4.

The measured axial stress–axial strain curves of the prepared specimens are plotted in Figure 11. Table 4 summarizes the parameters that represent the tensile properties of specimens, such as the initial cracking strain, ε_C,_ ultimate tensile strain, ε_U_, initial cracking stress, σ_C_, and tensile strength, σ_U_. The initial cracking stress and strain, ε_C_ and σ_C_, are the values of stress and strain at which appears the first crack on the specimen surface. The analysis of the uniaxial tensile tests of the studied specimens is shown in Figure 12.

The curves illustrate characteristics of multi-crack cracking in the EC0, EC1 and EC2 specimens and strain-hardening within EC2, EC3 and EC4 specimens, which exhibit a substantial ultimate tensile strain exceedingly over 2.5% (for EC4), 4.8% (for EC2) and 17.29% (for EC3), respectively. The ultimate tensile strength of the EC1, EC3 and EC4 were recorded as 11.76, 15.81 and 14.07 MPa, respectively. These results represent enhancements of 22.11, 64.17 and 46.1% over the tensile strength of EC0 standard (expired cement without composites considered as control sample). This under scored the significant improvement in tensile strength achieved by substituting expired cement with composite mixtures. The introduction of suitable composites enhances the ability of the cement matrix to absorb the tensile stress, leading to the improved mechanical performance of the composite–cement material.

**Figure 13:**
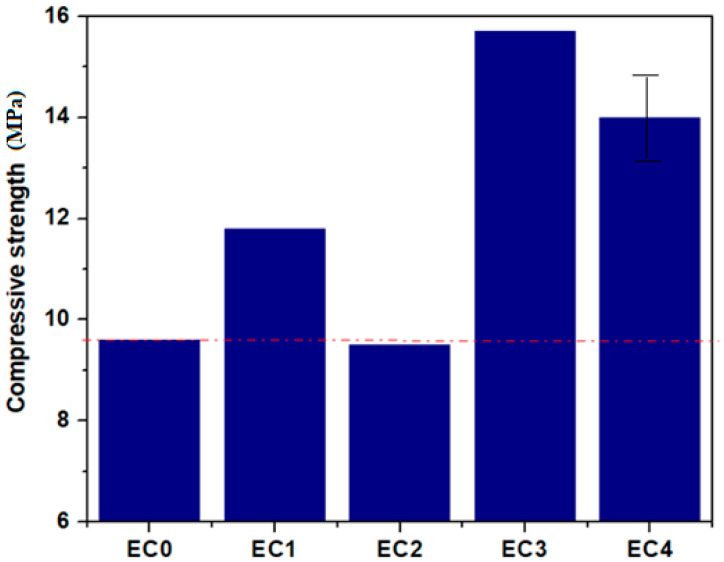
The compressive strength of matrix and mixtures with composites. The red line shows the compressive strength value of control sample.

The compressive strength results of the standard specimen and mixture specimens are illustrated in Figure 13. The measurements of the compression test are described in Refs. [28,29].

The reference group—EC0 specimen containing only expired cement—has a compressive strength of 9.6 MPa. The compressive strengths of specimen EC2 decreased, while for EC1, EC3 and EC4, the values improved comparatively with the EC0 standard etalon. When comparing the compressive strengths of EC1, EC3 and EC4, values of 11.8, 15.7 and 14 MPa were obtained, respectively. This indicates that the substitution of 2.5% expired cement with composites originating from construction and demolition enhanced the compressive strength of the standard specimen by over 22.9% for composites with lime, mortar and plaster, 63.5% for composites with iron, lead, steel iron and ash, and 45.8% for composites with nine waste in their structure, namely lime, mortar, plaster, ACC, brick, iron, lead, steel iron and ash. A replacement of expired cement with composites containing lime, mortar, plaster, brick and ACC reduces the compressive strength of the matrix by over 1%. The key factors contributing to the increase in compressive strengths of the cement matrix were the average crystallite sizes of particles in the composite structure and the formation of supplementary hydration products. The particle size of L, P and M composites was considerably smaller than that of A and B composites and, as a result, act as a fine aggregate that is more efficient for filling harmful pores within the expired cement matrix. In addition, the formation of new hydration products also contributes to the filling of the pores, and consequently, an enhancement in the strength of the cement matrix was achieved. Then, it is evident that a mixture containing 2.5% composite with metallic waste and ash exhibits notably higher compressive strength when compared to the matrix alone. This major enhancement in strength can be attributed to the suitable bridging effect within the cement matrix. Composites containing brick and ACC reduce the compressive strength of the expired cement matrix.

Our results show the feasibility of using a mixture composite as a substitute for cement in three formulations of the construction and demolition waste mix, namely EC2, EC3 and EC4 specimens.

In brief, an alternative to the conventional preparation of cement materials is the option of replacing them with fine composites containing construction and demolition waste. The acquisition of waste from construction companies does not involve financial expenses and yields a financial gain because they imply disposal fees.

## 4. Conclusions

In this study, the synthesis and characterization of new cement material with 2.5% nano-composites were discussed in view of applications as substitutes for cement in the constructions. Recycled powders from construction and demolition waste were used as raw materials in the preparation of the composites. These included recycled powders resulting from construction and demolition waste, namely broken glasses, lime, plaster, mortar, ACC and brick. The prepared nanocomposites were characterized by XRD, SEM, IR, UV–Vis and gap energy data. The X-ray diffractograms reveal the presence of CaCO_3_, SiO_2_, CaSO_4_ and CaAl_2_Si_2_O_8_ crystalline phases in the composites, depending on the waste types. The smaller particle sizes were found for the composites with lime, plaster and mortar.

The position of the IR band centered at approximately 985 cm^−1^ shifts to higher wave-numbers, suggesting the rearrangements in the silicate network and the formation of secondary C-S-H gel. The addition of the limestone composites in the standard cement led to an increase in the values of Vickers hardness.

Our results show the positive effect of the composite–cement material because the content of C-S-H gel increased the strength and stability of the cement paste.

This study also discussed the effect of nano-composites on waste cement. The conclusions can be drawn as follows: (i) the amount of C-S-H gel is almost the same as the standard cement control for the addition of lime composite in the expired cement mix; (ii) the values of the Vickers hardness increase after the replacement of the expired cement with lime or plaster composites.

The utilization of mixture composites as a partial replacement for expired cement led to a notable improvement in the compressive strength of the cement matrix due to the formation of new hydration products and the filling effect of fine composites. A positive impact on strain, tensile strength and compressive strength was observed in varying types of waste from the composite structures. In particular, compared with control expired cement, the tensile strength and compressive strength were increased by 2.5% and 45.83%, respectively, by replacing 2.5% expired cement with nanocomposites containing ten construction and demolition waste, namely glass, lime, mortar, plaster, ACC, brick, iron, steel iron, lead and ash powders.

The incorporation of various types of waste into expired cement matrix exhibits significant potential for improving mechanical properties and economic viability. The observed enhancement in strength, coupled with economic advantages, positions new composites-based recycled waste as promising materials for construction applications and practices.

## Figures and Tables

**Table 1:** Median particle size (D) of the high-intensity peak of prepared limestone–silicate composites.

Notation of Limestone–Silicate Composite	Composition Description of Limestone–Silicate Composite	Bragg Angle Corresponding to the Main Crystalline Phase 2θ (Degree)		β (Radian·10^−3^)	Median Particle Size for Peak D (nm)
L	Mixture of glassy (G) and lime waste	29.48	CaCO_3_	2.61	63.71
		34.22	Ca(OH)_2_	3.14	55.74
P	Mixture of glassy and plaster waste	29.48	CaCO_3_	1.39	119.63
		14.63	CaSO_4_	1.74	85.99
M	Mixture of glassy and mortar waste	26.73	SiO_2_	1.04	155.85
		29.48	CaCO_3_	1.74	95.55
A	Mixture of glassy and ACC waste	26.73	SiO_2_	1.22	132.88
		29.53	CaCO_3_	1.39	119.70
B	Mixture of glassy and brick waste	26.66	SiO_2_	0.87	186.20
		26.62	CaAl_2_Si_2_O_8_·4H_2_O	1.04	155.71
		36.5	CaCO_3_	0.69	261.00
Fiji Film images of the prepared samples: 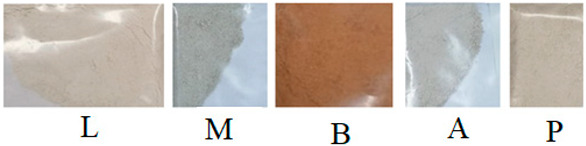					

**Table 2:** Description and notation of composite–cement materials.

Notation of Composite–Cement Materials	Description
Composite–cement probes with dimensions~3 × 2 × 1 cm	
C	cement material (using 100% validated cement)
CL	2.5%weight of validated cement are substituted by L, M, P, A or B composites
CM	
CP	
CA	
CB	
EC	expired cement material (using 100% expired cement)
ECL	2.5%weight of expired cement are substituted by L, M, P, A or B composites
ECM	
ECP	
ECA	
ECB	
Mixed composites–expired cement specimens having dimensions ~5 × 5 × 5 cm	
EC0	100% expired cement material
EC1	2.5%weight of expired cement are substituted by L, M, P, A and B mixed composites
EC2	2.5%weight of expired cement are substituted by L, M, P mixed composites
EC3	2.5%weight of expired cement are substituted by mixed composites containing iron, lead, cash iron and ash waste
EC4	2.5%weight of expired cement are substituted by L, M, P, A and B mixed composites and mixed composites containing iron, lead, cash iron and ash waste

**Table 3:** The test results of the studied samples: *P_max_*—maximum value of the compressive force on the sample, *R_c_*—compressive strength of the sample.

Sample Designation		Test Results	
		*P_max_* (kN)	*R_c_* (MPa)
TP4	EC1	29.2	11.8
TP5	EC2	22.1	9.5
TP6	EC3	38.1	15.7
TP7	EC4	34.0	14.0
Expired cement	EC0	22.9	9.6

**Table 4:** Summary of tensile parameters.

Specimen	Initial Cracking Strain, ε_C_(%)	Initial Cracking Stress, σ_C_ (Mpa)	Ultimate Tensile Strain, ε_U_(%)	Tensile Strength, σ_U_ (MPa)
EC0	2.54	6.72	3.47	9.63
EC1	1.64	6.5	2.82	11.76
EC2	2.02	5.54	3.64	9.53
EC3	3.08	7.98	4.07	15.81
EC4	3.07	7.96	3.56	14.07

## Data Availability

Data are contained within the article.
